# Single-cell transcriptomic insights into chemotherapy-induced remodeling of the osteosarcoma tumor microenvironment

**DOI:** 10.1007/s00432-024-05787-2

**Published:** 2024-07-20

**Authors:** Xuejing Zheng, Wence Wu, Zhenguo Zhao, Xinxin Zhang, Shengji Yu

**Affiliations:** 1 Departments of Orthopedics, National Cancer Center/National Clinical Research Center for Cancer/Cancer Hospital, Chinese Academy of Medical Sciences and Peking Union Medical College, No. 17 Nanli, Panjiayuan, Chaoyang District, Beijing, 100021 China; 2 Department of Orthopedics, Peking University First Hospital, Beijing, 100021 China

**Keywords:** Osteosarcoma, Chemotherapy, Single-cell sequencing, Tumor microenvironment, Cancer-associated fibroblasts

## Abstract

**Purpose:**

Neoadjuvant chemotherapy serves as an effective strategy for treating osteosarcoma (OS) not only by targeting cancerous cells but also by influencing the tumor's immune and stromal elements. Gaining insights into how chemotherapy reshapes the tumor's local environment is crucial for advancing OS treatment protocols.

**Methods:**

Using single-cell RNA sequencing, this study analyzed tumor samples from patients with advanced osteosarcoma collected both before and after chemotherapy.

**Results:**

The results revealed that chemotherapy caused the remaining OS cells to express higher levels of genes associated with stemness. Additionally, this process enhances the presence of cancer-associated fibroblasts, increasing their ability to modify the extracellular matrix (ECM). Chemotherapy also increases the number of endothelial cells, albeit with compromised differentiation capabilities. Importantly, the treatment reduced the immune cell population, including myeloid and T/NK cells, particularly impacting the subpopulations with tumor-fighting capabilities.

**Conclusion:**

These findings highlight the complex reaction of the tumor environment to chemotherapy, providing valuable insights into how chemotherapy influences OS cells and the tumor microenvironment (TME). This knowledge is essential for understanding OS resistance mechanisms to treatments, potentially guiding the development of novel therapies for managing advanced OS.

**Supplementary Information:**

The online version contains supplementary material available at 10.1007/s00432-024-05787-2.

## Introduction

Osteosarcoma typically manifests as spindle cell tumors that give rise to malignant osteoid tissue (Klein and Siegal 2006). It is widely believed to originate from the malignant transformation of mesenchymal lineage cells at an indeterminate stage of differentiation toward osteoblasts. This malignancy predominantly affects children and young adults, with the highest incidence occurring between the ages of 10 and 25 years (Mirabello et al. 2009). Osteosarcoma ranks as the most prevalent primary malignant bone tumor, characterized by its high aggressiveness, primarily affecting long bones in the extremities, such as the arms or legs; however, it can also manifest in other skeletal locations (Bielack et al. 2023). Standard treatment for osteosarcoma typically combines chemotherapy, surgical resection of the affected bone and adjacent tissue, and radiation therapy (Beird et al. 2022). However, in some cases, chemotherapy resistance develops, posing challenges to treatment efficacy (Lilienthal and Herold 2020). Overcoming chemotherapy resistance presents a formidable obstacle in osteosarcoma management, emphasizing the urgent need for novel strategies to enhance therapeutic outcomes for patients afflicted with this malignancy.

Single-cell RNA sequencing (scRNA-seq) is an advanced technology enabling the scrutiny of individual cell gene expression profiles, offering a greater degree of precision and intricacy than conventional bulk RNA sequencing techniques (Kolodziejczyk et al. 2015). Zhou et al. elucidated the landscape of intratumoral heterogeneity and the immunosuppressive microenvironment in advanced osteosarcoma following chemotherapy (Zhou et al. 2020). Liu et al. presented the single-cell atlas of treatment-naive osteosarcomas, illustrating the foundational state of these tumors (Liu et al. 2021). Although recent advancements in single-cell studies have illuminated aspects of osteosarcoma biology, a critical gap remains in understanding the specific impacts of chemotherapy on the tumor microenvironment (TME). Therefore, analyzing the changes in the TME before and after chemotherapy could be instrumental in understanding osteosarcoma drug resistance and identifying viable treatment strategies. However, research in this domain is currently lacking. Consequently, we analyzed the changes in the osteosarcoma TME before and after chemotherapy.

In this investigation, we used scRNA-seq to determine the heterogeneity of the TME in OS both pre- and post-chemotherapy. Through the scrutiny of gene expression profiles at the single-cell level, we discerned the intricate molecular pathways underlying chemotherapy resistance. This breakthrough allows us to develop novel therapies tailored to selectively target these mechanisms and overcome resistance. Such an approach holds promise for bringing about a paradigm shift in our comprehension of chemotherapy resistance, ultimately enhancing the prospects of patients with osteosarcoma.

## Results

### Single-cell analysis reveals the transcriptomic landscape in osteosarcoma of treatment-native and post-chemotherapy tissues

To investigate the heterogeneity of osteosarcoma between treatment-naive patients and those who underwent neoadjuvant chemotherapy, single-cell RNA sequencing data were obtained from six primary treatment-naive osteosarcomas (GSE162454 (Liu et al. 2021); patients_OS1/_OS2/_OS3/_OS4/_OS5/_OS6) and seven primary osteosarcomas post neoadjuvant chemotherapy (GSE152048 (Zhou et al. 2020); patients_BC2/_BC3/_BC5/_BC6/_BC16/_BC21/_BC22) from the GEO database (https://www.ncbi.nlm.nih.gov/geo/). Detailed clinical information for all patients is provided in Supplementary Table 1. After conducting quality control assessments based on nFeature_RNA, nCount_RNA, and the percentage of mitochondrial content (Fig S1A), a total of 97,416 cells were selected for further analysis, with an average of 2580 genes detected. The Seurat package (Zhang et al. 2018) was used for the analysis of the scRNA data, and the Harmony was applied to integrate samples to mitigate batch effects (Korsunsky et al. 2019).

**Fig. 1:**
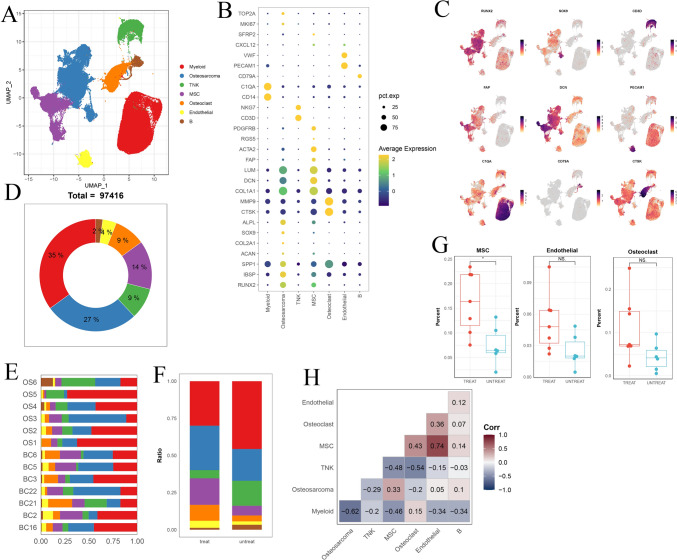
**A** UMAP plot depicting the distribution of 97,416 single cells categorized by major cell types. **B** Dot plot illustrating the expression of well-known marker genes within major cell types. **C** Feature plot highlighting the expression of recognized marker genes within major cell types. **D** Pie charts showing the proportions of various cell types. **E** The distribution of different cell types across patient samples. **F** Comparison of the composition of distinct cell types post-chemotherapy versus pre-chemotherapy osteosarcoma (OS) samples. **G** Box plot displaying the variations in MSCs, endothelial cells, and T/NK cell clusters between post- and pre-chemotherapy OS samples. **H** Pearson correlation analysis indicating the relationships among major cell types within OS

Unbiased clustering of all cells revealed seven main clusters in parallel, as determined by uniform manifold approximation and projection (UMAP) analyses based on their gene expression profiles and canonical markers (Fig. 1A–C). These clusters include osteosarcoma cells (n = 26,514) characterized by the expression of RUNX2, ALPL, IBSP, ACAN1, SOX9, and COL2A1 (Zhou et al. 2020); T/ILC cells (n = 8873) expressing the T-cell receptor (TCR) signaling mediators CD3E and CD3G (Zhang et al. 2018); B cells (n = 1878) marked by MS4A1 and CD79A (Martin et al. 2019); myeloid cells (n = 34,032) positive for CD14 and FCGR3A expression (Cheng et al. 2021); endothelial cells (ECs; n = 3868) identified by PECAM1 and CDH5 (Martin et al. 2019); osteoclast cells (n = 8352) marked by CTSK and MMP9 (Aliprantis et al. 2008); and mesenchymal stromal cells (MSCs; n = 13,899) identified by COL1A1 and COL3A1 (Martin et al. 2019). Among all the identified subgroups, myeloid cells constituted the largest proportion, accounting for approximately 35%, followed by osteosarcoma cells at 27%, with the smallest fraction being B cells at 2% (Fig. 1D). UMAP profiles and cell type contextures were stratified before and after neoadjuvant chemotherapy, facilitating the transcriptional comparison of corresponding cell populations in distinct clinical settings (Fig. S1B). Each patient sample contributed to the major cell type clusters, with B cells showing a slight dominance by OS6 (Fig. 1E, Fig S1C).

Although almost all cell clusters were present in all samples, the distribution of each cell type was not uniform across the specimens, indicating the heterogeneity of OS (Fig. 1E). Significantly, an increase in the presence of MSCs, endothelial cells, and osteoclasts was observed in post-chemotherapy tissues, suggesting their potential involvement in the tumor response to chemical agents (Fig. 1F, G; Fig. S1D, E). In contrast, immune cells, particularly T/NK lymphocytes, tended to be more prevalent in untreated osteosarcomas (Fig. S1D, E). These findings may indicate that chemotherapy promotes mesenchymal cell and endothelial cell infiltration but results in an immunosuppressive tumor microenvironment. As expected, correlation analysis revealed that MSCs and ECs, as well as T/NK and B cells, exhibited the most significant correlations. Cluster correlation analysis further demonstrated that MSCs and endothelial cells exhibited strong positive correlations across all clusters, whereas osteosarcoma displayed the most positive correlation with MSCs and had the greatest negative correlation with myeloid cells (Fig. 1H). This implies that both the antitumor and protumor subgroups undergo concurrent changes within their respective subpopulations, but these changes occur in opposite directions. In general, this may reflect the chemotherapy-induced evolution of subpopulations that promote osteosarcoma cells while suppressing those with antitumor properties. Given the heterogeneity within each subgroup, we further investigated the dynamic evolution of each subgroup.

### Dynamic changes in osteosarcoma cells before and after chemotherapy

**Fig. 2:**
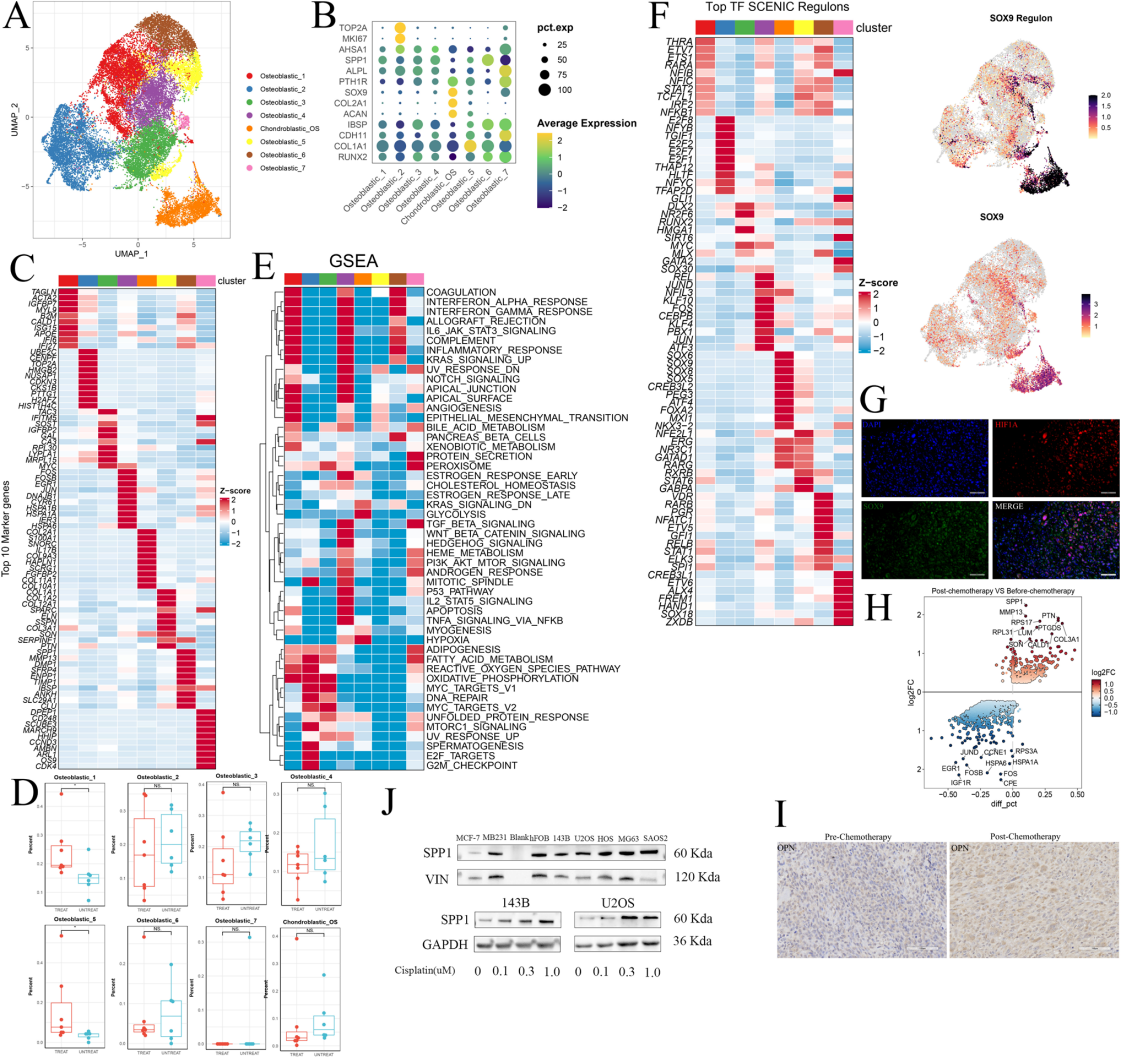
**A** UMAP plot showing seven osteoblastic osteosarcoma samples and one chondroblastic OS subclusters. **B** Dot plot representation of marker genes within each OS subgroup. **C** Heatmap displaying the top 10 marker genes specific to each OS subgroup. **D** Box plot illustrating the differential distribution of each OS subgroup between post- and pre-chemotherapy OS. **E** GSEA scores for hallmark gene sets in the MSigDB among each OS subgroup. **F** Heatmap of the top transcription factors (TFs) associated with OS calculated based on PySCENIC subgroups. Transcription factor regulon and corresponding SOX9 expression, indicating its regulation of OS by chondroblastic cells. **G** Representative images of immunofluorescence coexpression of HIF1A and SOX9 in osteosarcoma tissues (n = 3 from the National Cancer Center). **H** Differentially expressed genes (DEGs) between post- and pre-chemotherapy OS cells, with SPP1 upregulation. **I** Representative images of IHC staining of OPN in osteosarcoma pre- and post-chemotherapy tissues (n = 5 pairs from the National Cancer Center). **J** Western blot analysis of SPP1 expression in cell lines, including MCF7, MB-231, hFOB1.19, 143B, U2OS, HOS, MG63, and Saos2, with upregulated OPN expression in 143B and U2OS cells treated with cisplatin

In the clinic, osteoblastic and chondroblastic osteosarcoma (OS) are acknowledged as the two major primary subtypes of conventional OS. Using UMAP analysis of malignant OS cells, we identified a total of seven subclusters, six of which were associated with the osteoblastic lineage, whereas one was affiliated with the chondroblastic lineage (Fig. 2A; Fig. S2A, B, C). The gene expression patterns of OS-related genes within distinct clusters of malignant osteoblastic and chondroblastic OS cells are depicted in Fig. 2B and Fig. S2C. The osteoblastic 1 cluster exhibited the characteristic expression of TAGLN and IGFBP7, and the osteoblastic 2 cluster exhibited the expression of TOP2A and CDKN3, which is indicative of a proliferating OS cluster. The osteoblastic 4 cluster was characterized by the expression of FOS, JUN, and HSPA1A, indicating its role in the stress response (Fig. 2C).

The distribution of each OS cluster from various patients or between treatment-naïve and post-chemotherapy lesions exhibited significant variation, underscoring the heterogeneity of advanced OS, as depicted in Fig. S2D, E. Notably, the osteoblastic_1 cluster, representing the major type of osteosarcoma cell and accounting for 35% of all osteosarcoma cells, showed a substantial increase in the chemotherapy group, suggesting its potential responsiveness to chemotherapy (Fig. 2D). To elucidate the underlying biological alterations within this subcluster, we conducted an analysis of hallmark gene sets from the Molecular Signatures Database (MsigDB) (Liberzon et al. 2015) and identified upregulated pathways associated with EPITHELIAL_MESENCHYMAL_TRANSITION and ANGIOGENESIS in the hallmark of the osteoblastic_1 cluster (Fig. 2E). This common evidence indicates that OS cells undergo progression when subjected to chemotherapy, leading to enhanced epithelial–mesenchymal–transition (EMT) and increased endothelial cell proliferation.

BC22, a chondroblastic osteosarcoma, is the largest tumor measuring 18 × 15 × 12 cm and displays a necrosis rate of less than 90% after four rounds of neoadjuvant chemotherapy (Zhou et al. 2020). The necrotic rate of osteosarcoma is determined by pathologists. According to the Huvos criteria, grade 1 equates to less than 50% necrosis, grade 2 is 50–90%, grade 3 is 90–99%, and grade 4 is 100% necrosis (Luetke et al. 2014). As anticipated, the hypoxia and glycolysis pathways were the most activated pathways among all the tumors (Fig. 2E). Notably, HIF1A serves as the hallmark gene for hypoxia, and previous studies have reported that HIF1A, a critical gene within the hypoxia pathway, activates SOX9 as a transcription factor in chondroblastic osteosarcoma (Shao et al. 2021). This led us to investigate whether SOX9 transcriptional activity was indeed upregulated. We computed the transcription factors (TFs) for each cluster using pySCENIC (Sande et al. 2020), and the results clearly demonstrated that Chondroblastic_OS was primarily regulated by the SOX9 regulon, as also evidenced by its elevated SOX9 expression levels (Fig. 2F). Immunofluorescence (IF) coexpression of HIF1A and SOX9 (n = 3, sourced from the National Cancer Center) confirmed our hypothesis to some extent (Fig. 2G). These findings underscore the pivotal role of the SOX9 transcription factor in chondroblastic osteosarcoma.

Furthermore, our analysis of DEGs in OS tissues before and after chemotherapy revealed notable DEGs, such as SPP1, MMP13, FOS, and JUN (Fig. 2H). OPN is a pleiotropic protein encoded by SPP1 (Butti et al. 2021). Immunohistochemistry (IHC) confirmed the increased OPN protein expression in osteosarcoma cells after chemotherapy (Fig. 2I, Fig. S2F, n = 5 per group, sourced from the National Cancer Center). The Gene Ontology biological process analysis of DEGs revealed enrichment in processes related to oxidative phosphorylation and epithelial–mesenchymal transition (EMT), further substantiating the impact of chemotherapy on the induction of EMT in osteosarcoma cells (Fig. S2G). Given that SPP1 was one of the most significantly altered DEGs, we sought to validate its role in the chemotherapy response. We examined SPP1 expression in osteosarcoma cells, with MCF7 serving as a negative control and MB231 serving as a positive control for SPP1 expression, as previously reported. Notably, we analyzed SPP1 protein levels in several osteosarcoma cell lines, including 143B, MNNG, U2OS, HOS, SAOS2, and MG63. Surprisingly, all these osteosarcoma cell lines exhibited SPP1 expression (Fig. 2J). Subsequently, we subjected osteosarcoma cell lines (143B and U2OS) to cisplatin, a commonly used chemical drug. As anticipated, SPP1 was upregulated in response to cisplatin treatment in both cell lines in a dose-dependent manner (Fig. 2J). These compelling findings strongly suggest that SPP1 may indeed play a critical role in imparting chemoresistance to cancer cells.

Additionally, we examined copy number variation (CNV) within sarcoma cells using the "infercnv" package. Surprisingly, we observed fewer CNVs in post-chemotherapy tissues, contrary to our initial expectations (Fig. S2H). Our hypothesis posits that chemical agents may selectively target osteosarcoma cells with a higher CNV, ultimately allowing cells with a lower CNV to survive and thereby contributing to chemotherapy resistance. In conclusion, our study provides insights into the dynamic changes in osteosarcoma cells in response to chemotherapy, shedding light on potential therapeutic targets and mechanisms of resistance.

### Alterations in mesenchymal stromal cells before and after chemotherapy in osteosarcoma

Building on our previous investigations into the changes observed in OS tumor cells before and after chemotherapy, we now shift our focus toward studying the dynamic alterations in mesenchymal stromal cells. As crucial components of the tumor microenvironment, mesenchymal stromal cells play pivotal roles in tumor growth, invasion, and response to therapy. This subsequent study aimed to elucidate the molecular and functional changes occurring in mesenchymal stromal cells during chemotherapy in osteosarcoma patients, shedding light on their potential impact on tumor progression and treatment outcomes.

**Fig. 3:**
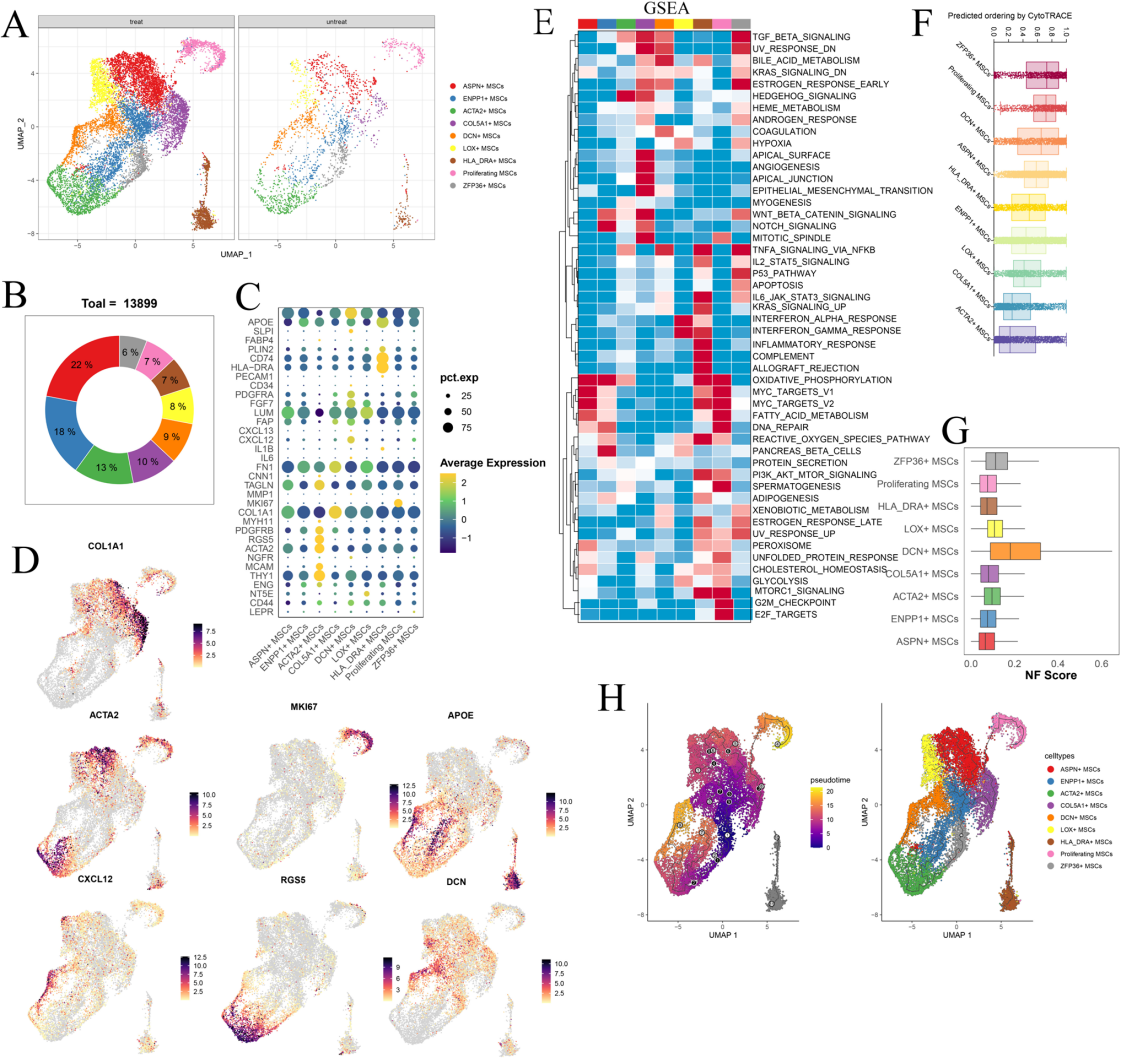
**A** UMAP plot illustrating the distribution of major mesenchymal stromal cells (MSC) subgroups in post- and pre-chemotherapy samples. **B** Pie charts displaying the percentage distribution of different MSC subgroups. **C** Dot plot representation of marker genes within each MSC subgroup. **D** Feature plot of marker genes within each MSC subgroup. **E** GSEA scores of each MSC subgroup for hallmark gene sets in MSigDB. **F** Cytotrace analysis highlighting the ZFP36 + MSCs with the highest stemness score. **G** AUCell analysis revealing that DCN + MSCs have the highest signature score for normal fibroblasts (NFs). **H** Monocle3 trajectory analysis demonstrating the differentiation potential of ZFP36 + MSCs into ASPN + MSCs, ACTA2 + MSCs, and COL5A1 + MSCs

Within the 13,899 mesenchymal stromal cell population, we identified nine distinct cell subtypes based on specific markers: ASPN + MSCs (identified by the marker genes OGN and S100A13), ENPP1 + MSCs (marked by ANKH and ENPP1), ACTA2 + MSCs (characterized by ACTA2 and RGS5), COL5A1 + MSCs (expressing COL5A1 and COL16A1), DCN + MSCs (with the markers DCN and TXNIP), LOX + MSCs (marked by LOX and TGFBI), HLA_DRA + MSCs (distinct by HLA-DPA1 and C1QC), proliferating MSCs (determined by MKI67 and TOP2A), and ZFP36 + MSCs (identified by ZFP36 and JUN) (Fig. 3A–D; Fig. S3A, B, C). We then applied gene set enrichment analysis to uncover hallmark pathways that were enriched for each cell type (Fig. 3E). Notably, COL5A1 + MSCs displayed the most pronounced signature related to angiogenesis and epithelial–mesenchymal transition (EMT). Cytotrance analysis revealed that ZFP36 + MSCs exhibited the highest stem score, suggesting that they might serve as the source of all MSCs. Conversely, LOX + MSCs, COL5A1 + MSCs, and ACTA2 + MSCs exhibited the lowest stem scores, suggesting that they were highly differentiated cells (Fig. 3F). DCN, a marker for normal fibroblasts, demonstrated the highest signature score for normal fibroblasts among DCN + MSCs (Fig. 3G). Monocle3 analysis provided evidence for a differentiation trajectory originating from ZFP36 + MSCs to LOX + MSCs, COL5A1 + MSCs, and ACTA2 + MSCs (Fig. 3H).

At the global level, we conducted a differential gene expression analysis of MSCs in osteosarcoma tissues before and after chemotherapy. Our analysis revealed that the most significantly upregulated genes were related to the extracellular matrix (ECM), particularly to TIMP1 and TIMP3, as well as to CXCLs (Fig. S3D). Notably, among all the cell clusters, COL5A1 + MSCs were increased in osteosarcoma tissues following chemotherapy (Fig. S3E). A heatmap of the differentially expressed genes illustrated the elevated expression of genes such as COL5A1, COL16A1, EMILIN1, and ADAMTS2 in this cluster (Fig. S3F). These genes are primarily associated with ECM remodeling, suggesting that MSCs may contribute to the reconfiguration of the ECM in response to chemotherapy, consistent with our previous findings from hallmark gene set enrichment analysis. Targeting this specific cluster of MSCs holds promise for enhancing osteosarcoma treatment.

In this section, we undertake a more in-depth exploration of the characteristics of mesenchymal cells within the osteosarcoma microenvironment. We aimed to comprehensively understand their responses to chemotherapy and elucidate the underlying mechanisms that govern their behavior. By deciphering the complex interplay between osteosarcoma cells and stromal cells in their vicinity, our goal is to uncover valuable insights into the intricate crosstalk within the tumor microenvironment and identify potential therapeutic targets.

### Dynamic changes in endothelial cells within the osteosarcoma microenvironment before and after chemotherapy

**Fig. 4:**
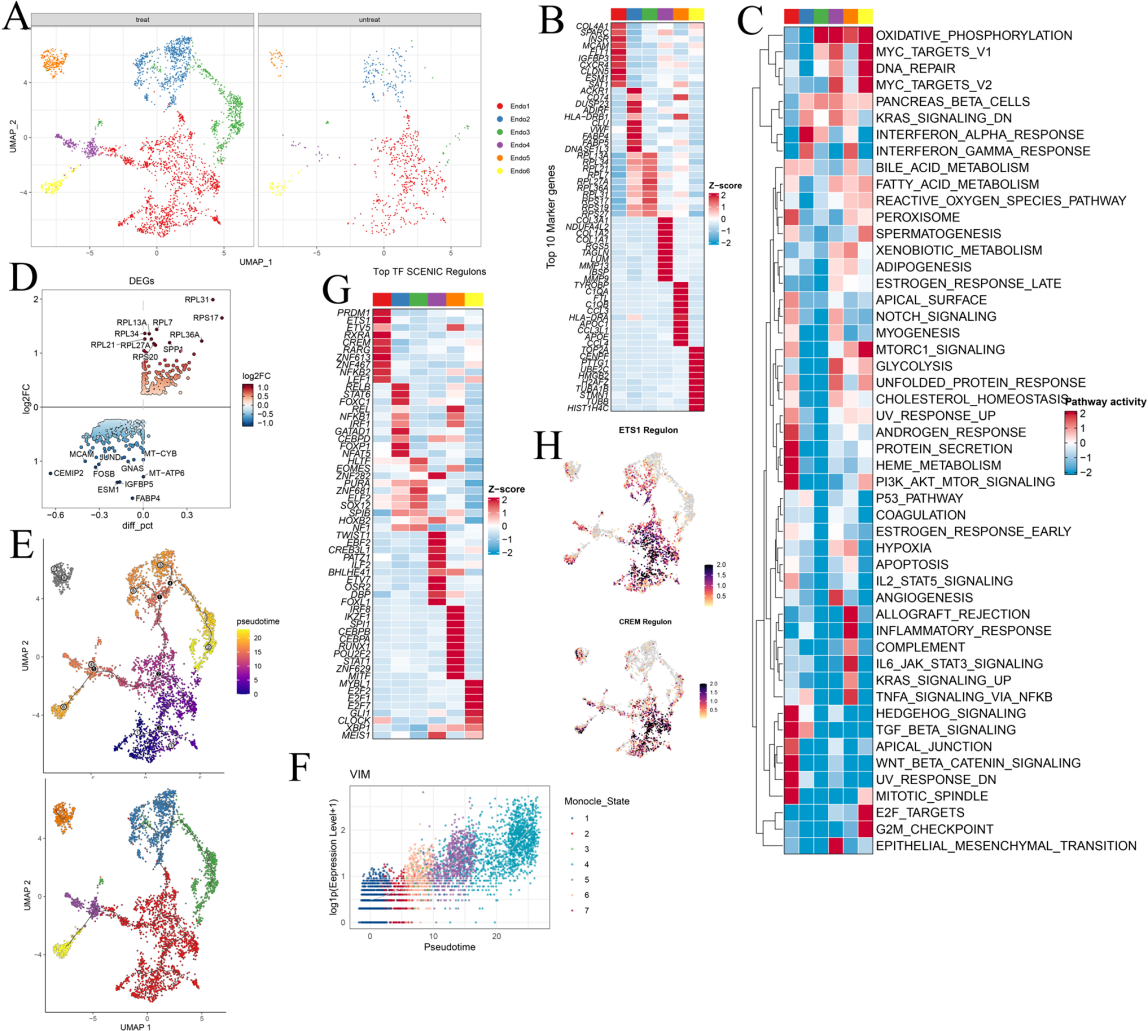
**A** UMAP plots illustrating the distribution of major endothelial cell subgroups in post- and pre-chemotherapy samples. **B** Heatmap displaying the top 10 marker genes among the endothelial subgroups. **C** GSEA scores of each endothelial subgroup for hallmark gene sets in MSigDB. **D** DEGs identified between post- and pre-chemotherapy osteosarcoma cells. **E** Monocle3 analysis showing the differentiation trajectory of endothelial subgroups. **F** VIM (Vimentin) was upregulated along the pseudotime trajectory. **G** Heatmap showing the top transcription factors (TFs) identified by PySCENIC among endothelial subgroups. **H** Transcription factor regulon analysis revealing that ETS1 and CREM regulate Endo1

In a previous study, it was reported that chemotherapy led to a reduction in the percentage of endothelial cells in esophageal cancer (Croft et al. 2022). However, the proportion of endothelial cells slightly increased within the tumor microenvironment in osteosarcoma (Fig. 1G). The endothelial cells were classified into six distinct clusters with specific markers (Fig. 4A, B; Fig. S4A–C). The proportion of each subgroup exhibited variations among different patients and groups (Fig. S4D, E). Using gene set enrichment analysis (GSEA), we identified the following distinct features of these endothelial cell clusters. Endo1 demonstrated high COL4A1 and SPARC expression and exhibited enrichment in pathways related to PROTEIN_SECRETION and TGF_BETA_SIGNALING (Fig. 4C). Endo2 displayed high ACKR1 and VWF expression and enrichment in pathways associated with INTERFERON_ALPHA_RESPONSE and INTERFERON_GAMMA_RESPONSE. Endo4 exhibited high LUM and MMP13 expression and enrichment in pathways associated with OXPHOS, angiogenesis, angiogenesis, and EMT. Endo5 expressed genes related to inflammatory and immune responses, such as C1QA, C1QB, CCL3, and HLA − DRA. Endo6 represented a proliferating subcluster with TOP2A and STMN1 expression, indicating its role in proliferation.

In general, chemotherapy induced high metabolic activity in endothelial cells, as indicated by the differential expression of the RPS and RPL genes (Fig. 4D). The cell proportion analysis also revealed an increase in Endo3 expression, which is characterized by RPS and RPL genes, in osteosarcoma patients post neoadjuvant chemotherapy, corroborating findings from the previous DEG analysis (Fig. S4F). Moreover, the expression of Endo4 increased. Based on the high expression of mesenchymal markers, such as COL1A1 and LUM, we hypothesize that these cells may represent a subpopulation of cells undergoing chemotherapy-induced endothelial-mesenchymal transition (Endo-Mesenchymal transition). This hypothesis aligns with the earlier GSEA results (Fig. 4B). Notably, Endo4 exhibited the highest signature for angiogenesis (Fig. 3C). Cytotrance analysis demonstrated that Endo1 cells had relatively high stemness (Fig. S4G), and Monocle 3 analysis revealed the potential of these cells to differentiate into other clusters during differentiation (Fig. 4E). The expression of VIM, a mesenchymal marker gene associated with epithelial–mesenchymal transition (EMT), increased during differentiation (Fig. 4F).

Endo1 was decreased post-chemotherapy, suggesting that chemotherapy may target endothelial stem cells and impede the formation of endothelial cells with normal functionality (Fig. S4). This led us to further investigate the transcription factor regulatory network. In the analysis of Endo1, pySCENIC highlighted the significance of ETS1 in endothelial cell development (Fig. 4G, H). ETS1 is one of the key members of the ETS transcription factor family and plays a crucial role in vascular endothelial cells by reactivating quiescent endothelial cells to enter a state of angiogenesis (Chen et al. 2017). Additionally, we identified NFKB2 and CREM as key players in endothelial cells. Further research into these transcription factors may provide insights into the impact of chemotherapy on endothelial cells.

### Alterations in osteoclasts during pre- and post-chemotherapy osteosarcoma

**Fig. 5:**
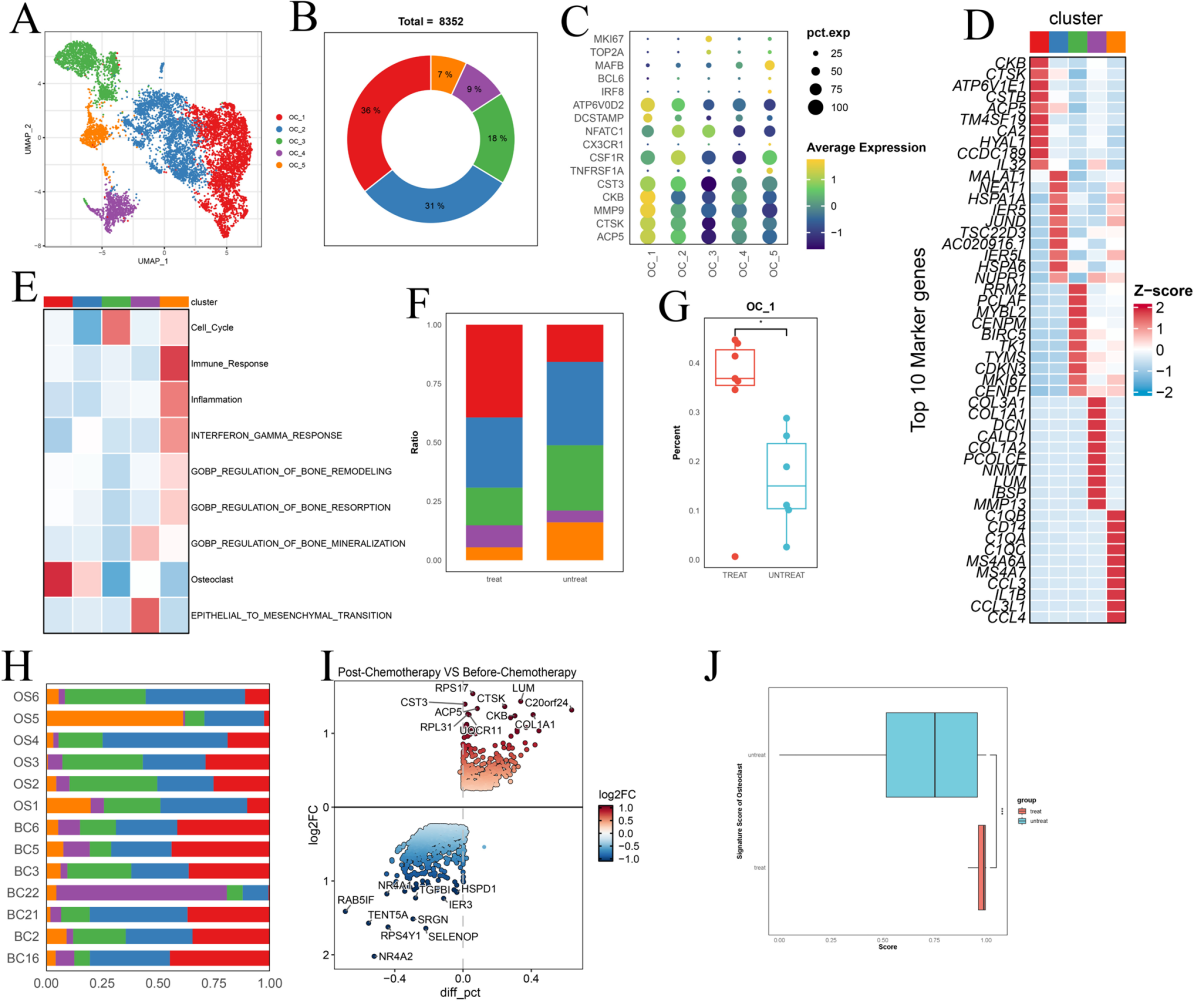
**A** UMAP plot displaying the distribution of each osteoclast subgroup. **B** Pie charts showing the percentages of patients in different OC subgroups. **C** Dot plot revealing marker genes in each OC subgroup. **D** Heatmap illustrating the top 10 marker genes among the OC subgroups. **E** AUCell scores indicating the signature of each OC subgroup. **F** Histogram showing the different compositions of OC subgroups between post- and pre-chemotherapy groups. **G** The proportion of OC_1 increased in post-chemotherapy OS patients. **H** Histogram displaying the different compositions of OC subgroups among patients. **I** DEGs identified between post- and pre-chemotherapy samples with upregulated ACP5 and CTSK expression. **J** GSVA scores indicating the enhanced activity of osteoclasts post-chemotherapy osteosarcoma

Osteoclasts, a specialized lineage of relatively large, multinucleated monocyte-macrophage cells, play a pivotal role in facilitating osteolysis and providing crucial support for tumor growth within osteosarcoma tissues (Akiyama et al. 2008). We identified 8352 osteoclasts based on their distinct expression of ACP5, CTSK, and MMP9 and subsequently categorized them into 5 distinct clusters (Fig. 5A, B). As illustrated in Fig. 5C, D and Fig. S1A, B, OC_1 exhibited notably high levels of mature osteoclastic markers, including CTSK, MMP9, and ACP5. OC_2 displayed the second-highest levels of these markers, indicating that it represents a subgroup of less mature osteoclasts. Conversely, OC_3 displayed the lowest ACP5 and CTSK expression but exhibited high levels of proliferation markers such as MKI67 and TOP2A. OC_5 expressed markers such as C1QA, C1QC, CCL3, and CCL4, indicating a potential role in the immune response. We confirmed these findings through gene set variation analysis (GSVA) (Fig. 5E).

The overall composition of osteoclasts exhibited notable differences among different patients (Fig. S5C). Furthermore, the overall composition of the osteoclast cell compartment underwent some degree of modification following chemotherapy (Fig. 5F). Most notably, a significant increase in the relative proportion of OC_1 was noted, with no such shift observed in other clusters (Fig. 5G, Fig. S5D). This suggests that chemotherapy may promote the maturation of osteoclasts, potentially enhancing their bone resorption activity and, consequently, the tumor's ability to locally invade surrounding tissues. PySCENIC analysis identified FOS as a major transcription factor of OC_1 (Fig. S5E), corroborating earlier findings (Axelrod et al. 2019). Targeting this transcription factor may help mitigate bone degradation by osteoclasts during chemotherapy. Notably, in the case of BC22, a chondroblastic osteosarcoma that underwent chemotherapy, as previously mentioned, it was predominantly composed of OS_4 (Fig. 5H). This finding underscores the heterogeneity of osteoclasts between osteoblastic and chondroblastic osteosarcomas. Interestingly, we found that SOX9 plays a significant role as a transcription factor in osteoclasts in chondrosarcoma. This transcription factor is also important in chondroblastic osteosarcoma cells. Regardless of the proportion of each cluster, we observed that pro-osteoclast signatures were consistently elevated in all clusters following chemotherapy, as evidenced by differentially expressed genes (DEGs), such as CTSK and ACP5, when comparing osteosarcomas post-chemotherapy with those before treatment (Fig. 5I). These findings were further validated by calculating the signature score of osteoclasts using AUCell (Fig. 5J). Collectively, these data suggest that chemotherapy enhances the activity of osteoclasts, potentially contributing to local tumor invasion.

### Changes in tumor-infiltrating lymphocyte subsets before and after chemotherapy in osteosarcoma

**Fig. 6:**
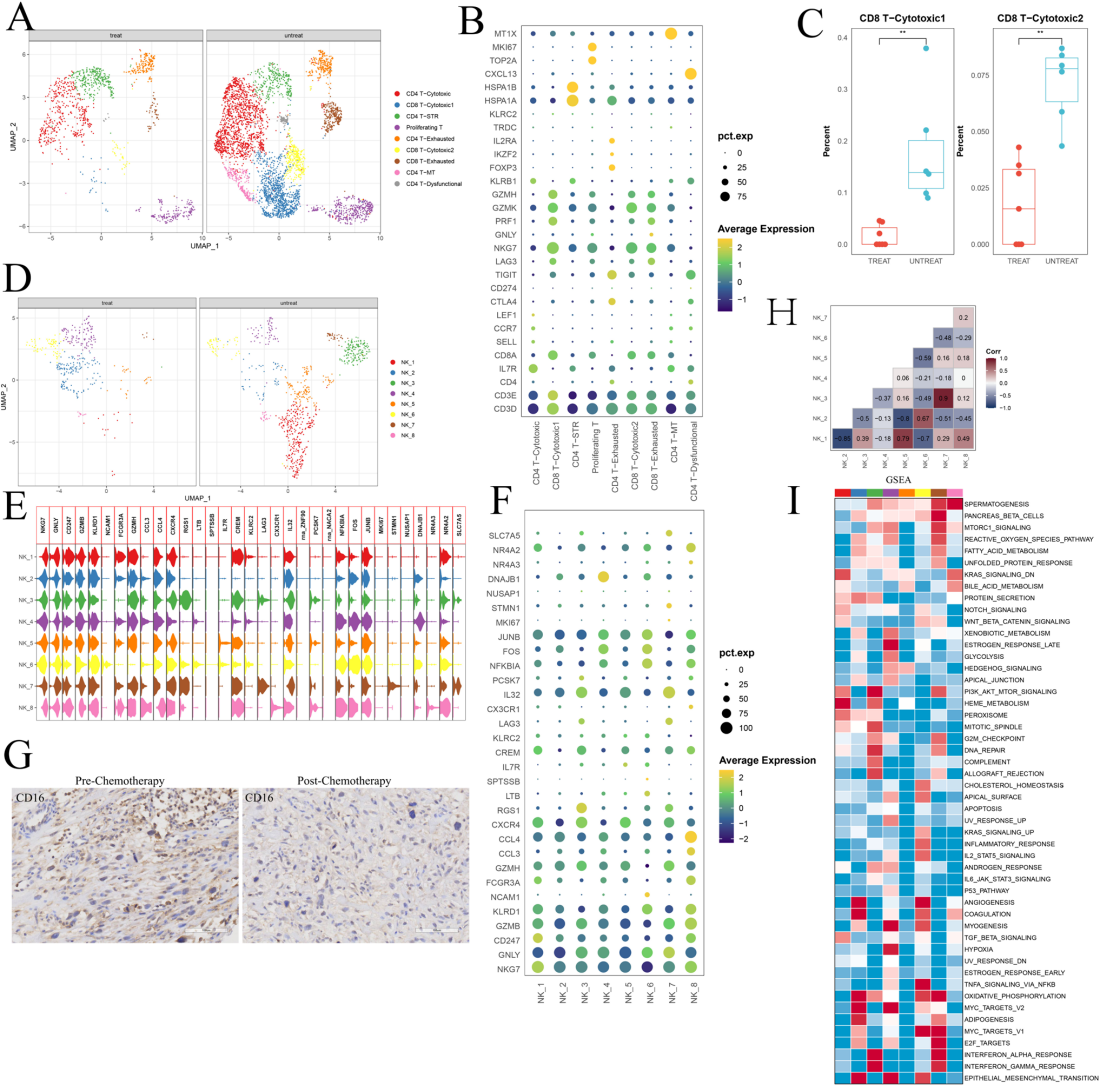
**A** UMAP plot showing the distribution of major T-cell subgroups between post- and pre-chemotherapy osteosarcoma samples. **B** Dot plot displaying marker genes in each T-cell subgroup. **C** Box plot indicating the decreased proportion of CD8 + T-cell cytotoxic subgroups in post-chemotherapy osteosarcoma samples. **D** UMAP plot illustrating the distribution of major NK cell subgroups between post- and pre-chemotherapy osteosarcoma samples. **E**, **F** Dot plot and violin plot visualizing markers in each NK subgroup. **G** Immunohistochemical analysis of CD16 in pre- and post-chemotherapy osteosarcoma samples (n = 5 pairs from the National Cancer Center). **H** Pearson correlation analysis showing the correlation between each NK subgroup. **I** GSEA scores of each NK subgroup indicating the hallmark gene sets from the MSigDB

To gain a more comprehensive understanding of TILs, we conducted an in-depth analysis of the T/NK cell population. Using unsupervised clustering of 25,588 T cells, which represented the dominant lymphocyte population, we identified nine distinct T-cell populations, comprising five CD4 + subtypes, three CD8 + subtypes, and one proliferating T-cell population (Fig. 6A; Fig. S6A, B). Within the CD8 + T-cell clusters, two cytotoxic subpopulations characterized by GZMK and GZMH expression were identified (Fig. 6B). Notably, both cytotoxic clusters exhibited downregulation in OS following chemotherapy (Fig. 6C). Notably, the composition of the T-cell subgroups varied among the individual patients (Fig. S6C).

Furthermore, the two CD8 + cytotoxic clusters demonstrated upregulation of NKG7, GZMB, GZMH, GZMK, CCL3, CCL4, and CCL5 (Fig. S6D), suggesting that chemotherapy amplified the cytotoxic function of CD8 + T cells, potentially enhancing their antitumor activity within the osteosarcoma tumor microenvironment. However, it is important to acknowledge that a reduction in CD8 + T cells was noted in post-chemotherapy osteosarcomas (Fig. S6E), which could imply a diminished antitumor capability. This finding reflects the intricate and multifaceted impact of chemotherapy on the immune response.

Our ongoing research is primarily focused on unraveling the dynamic alterations occurring within NK cell subpopulations before and after chemotherapy in the context of osteosarcoma. In various cancer types, NK cells are typically categorized into two main subsets, including CD56bright CD16low and CD56dim CD16high, which are characterized by their distinctive expression patterns of the canonical cell markers NCAM1 and FCGR3A (Fig. 6D, E, F) (Tang et al. 2023). In our investigation, we further stratified NK cells into eight subgroups, with six clusters (NK_1, NK_2, NK_4, NK_5, NK_7, and NK_8) falling within the CD16high category (as indicated by FCGR3A expression), whereas subgroup NK_6 pertains to the CD56high category (distinguished by NCAM1 expression). The CD16high subgroups exhibited specific gene signatures, including CREM (NK_1), RGS1 (NK_7), DNAJB1 (NK_4), CXCR4 (NK_5), IL32 (NK_7), and CCL4 (NK_8).

Our findings revealed a consistent prevalence of CD16high subgroups, characterized by FCGR3A expression, within the osteosarcoma microenvironment both before and after chemotherapy (Fig. 6D). As previously mentioned, there was an overall reduction in NK cells following chemotherapy, as evidenced by CD16 immunohistochemistry (Fig. 6G, Fig. S6F; n = 5 pairs from the National Cancer Center). Following chemotherapy, we observed an increased proportion of the NK_2 and NK_6 subpopulations, accompanied by a reduction in the proportion of the NK_1, NK_3, and NK_5 subgroups (Fig. S6G). Importantly, the remaining subgroups displayed marginal alterations. Additionally, using Pearson correlation analysis of the proportions of NK cell subpopulations in each patient, we identified correlations among subgroups (Fig. 6H). Specifically, the NK-1, NK_5, and NK_7 subgroups exhibited positive correlations, whereas the NK_2 and NK_6 subgroups displayed positive correlations. Intriguingly, subgroups NK_1, NK_5, and NK_7 showed negative correlations with subgroups NK_2 and NK_6, suggesting potential dynamic shifts in response to chemotherapy. This prompted us to explore the functional attributes of these subgroups. Using gene set enrichment analysis (GSEA), we conducted an in-depth analysis of the functional characteristics of these subgroups (Fig. 6I). We found that NK_2 cells were primarily enriched in pathways associated with oxidative phosphorylation and adipogenesis, whereas NK_6 cells were enriched in signaling pathways, such as angiogenesis, TNFA signaling via NFKB, and epithelial–mesenchymal transition. Among the subgroups with reduced expression post-chemotherapy, NK_1 was enriched in pathways related to heme metabolism and peroxisomes, whereas NK_3 was enriched in signaling pathways such as the interferon alpha response and interferon gamma response. NK_5 was enriched in pathways such as hedgehog signaling and bile acid metabolism.

In summary, our research suggested that chemotherapy may induce the transformation of relatively immune-enriched NK cells with tumor-inhibitory potential into NK cells that promote tumor progression. As mentioned earlier, this finding implies that chemotherapy leads to the development of an immune-suppressive tumor microenvironment.

### Modulation of myeloid cell populations in osteosarcoma following chemotherapy

**Fig. 7:**
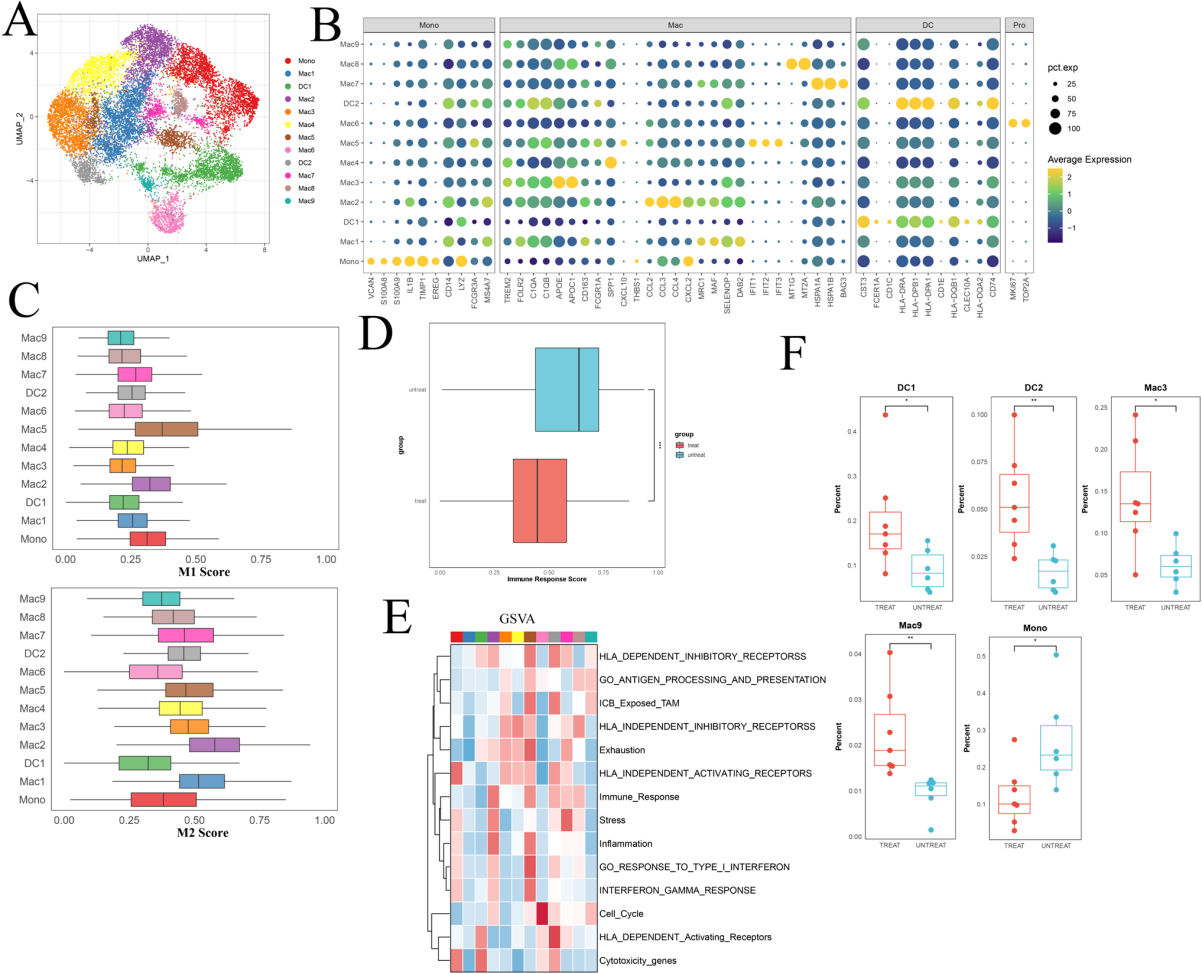
**A** UMAP plot depicting the distribution of major myeloid cell subgroups between post- and pre-chemotherapy osteosarcoma samples. **B** Violin plot illustrating marker genes in each myeloid cell subgroup. **C** AUCell scores indicating the M1 and M2 macrophage signatures among myeloid subgroups. **D** AUCell scores displaying the immune response signature among myeloid subgroups. **E** GSVA of specific signatures for each myeloid subgroup. **F** Box plot demonstrating the variation in myeloid subgroups between the post- and pre-chemotherapy groups

Given that the fraction of macrophages/monocytes in OS tissues decreased after chemotherapy (Fig. 1E), we investigated the composition and gene expression of subclusters within the myeloid cell population. Reclustering of all 15,340 myeloid cells revealed 12 distinct myeloid cell populations with varying frequencies in different tissues (Fig. 7A, B; Fig. S7A, B). The monocyte population exhibited high VCAN, S100A9, EREG, and LYZ expression. Additionally, we identified two clusters of classical dendritic cells (DCs) expressing CD1C, CST3, and HLA-DBP1 (Fig. 7B). Furthermore, we identified 9 macrophages expressing C1QA, APOE, and TREM2 (Fig. 7B). Notably, Mac5 expressed proinflammatory genes, such as IFIT1 and CXCL10, which may play a role in promoting antitumor immunity. To validate this finding, we utilized AUCell to assess its gene set signature (Fig. 7C). As demonstrated, Mac5 displayed the highest signature score for M1 macrophages, indicating its involvement in antitumor activities. Conversely, Mac2, a marker associated with protumor functions, exhibited the highest M2 signature score. These findings collectively suggest that the myeloid cell population comprises both protumor and antitumor subtypes.

The composition of myeloid cell subgroups varied among patients (Fig. S7C). Furthermore, a notable decrease in the proportion of monocytes (Mono) and an increase in the proportions of DC1, DC2, Mac3, and Mac9 cells were observed (Fig. 7B). We noted an increase in dendritic cells (DCs) in osteosarcoma post-chemotherapy (Fig. 7F), and this subgroup was characterized by cytotoxicity genes and HLA-dependent activating receptors. In contrast, the monocyte population decreased and exhibited a high signature of HLA-independent activating receptors. This underscores the complex impact of chemotherapy, resulting in a generally decreased immune response within the tumor microenvironment. The DEGs of monocytes between the chemotherapy and treatment-naive groups included stress-related genes such as FOS, JUN, HSP, and DNAJB1 (Fig. 7C). In summary, chemotherapy appears to promote a reduced immune response within the tumor microenvironment.

## Discussion

Bulk RNA-sequencing studies on OS patients post-chemotherapy, have revealed gene signature modifications and immune cell population shifts (Sun et al. 2023; Li et al. 2022). However, these analyses lacked the resolution to detail gene expression within specific cell subclusters. Our research, which focused on single-cell-level gene expression in osteosarcoma, suggested that chemotherapy could promote angiogenesis and stromal cell infiltration in the tumor microenvironment. Additionally, it may enhance osteoclast activity, aiding in local invasion of osteosarcoma cells. Given the documented chemotherapy resistance in osteosarcoma, our findings suggested that chemotherapy could intensify its malignancy.One of our most compelling findings revolves around the increased expression of SPP1 in post-chemotherapy osteosarcoma cells. SPP1 is associated with cancer stemness and is capable of driving cancer progression and metastasis (Sun et al. 2021). This observation suggested that chemotherapy may contribute to the survival of OS stem cells and promote their stemness. Furthermore, our data indicate that osteosarcoma cells may undergo epithelial–mesenchymal–transition, leading to chemotherapy resistance, particularly through the increased presence of Osteoblastic-1. EMT is widely acknowledged for its role in chemotherapy resistance and metastasis (Huang et al. 2022). This finding implies that osteosarcoma cells activate EMT as a defense mechanism against the detrimental effects of chemotherapy. Previous reports have highlighted that radiotherapy and chemotherapy can enhance the expression of MHC-II genes in cancer cells (Liu et al. 2023). Increased MHC-II expression in tumor cells has been associated with the efficacy of immune checkpoint inhibitors and a more favorable disease prognosis (Axelrod et al. 2019). This suggests that chemotherapy might enhance the potential of immune therapy. However, we did not observe significant variations in the expression of MHC-II genes, indicating that chemotherapy might not be conducive to promoting immune therapy in patients with osteosarcoma. This finding aligns with the clinical observation that osteosarcoma is considered an immunologically "cold" tumor that is often unresponsive to immune therapy (Chen et al. 2021). In parallel, we did not detect an accumulation of CD4 + or CD8 + T cells, further suggesting that immune therapy might not synergize with chemotherapy. Hence, future studies should prioritize exploring strategies to enhance MHC-II expression or increase the presence of CD4 + and CD8 + T cells in the context of osteosarcoma.

Cancer-associated fibroblasts (CAFs) constitute the predominant cellular component of the tumor microenvironment, and their prominence is underscored by their abundance and extensive crosstalk with cancer cells (Chen et al. 2023). This study revealed an increase in mesenchymal stromal cells. Considering their known role in bolstering tumor survival and progression (Fiori et al. 2019), we hypothesize that osteosarcomas that survive chemotherapy may engage in crosstalk with CAFs to further enhance their survival and progression. Consistent with the observed changes in cancer cells, alterations in the stromal components of the tumor microenvironment also indicate that chemotherapy could potentiate the malignant activity of osteosarcoma cells. These findings provide supporting evidence that if OS displays resistance to neoadjuvant chemotherapy, a transition to surgical intervention may be a reasonable alternative.

We also noted changes in the relative proportions and transcriptional characteristics of T and NK cell subclusters subsequent to chemotherapy. CD56dim CD16 + NK cells are typically considered more cytotoxic than CD56bright NK cells, given the ability of CD16 to mediate antibody-dependent cell-mediated cytotoxicity (Freud et al. 2017). Interestingly, in conjunction with the decrease in TNK cells, there appears to be a decrease in CD56dim CD16 + NK cells following chemotherapy, while CD56bright NK cells exhibit a contrasting trend. Furthermore, the percentage of cytotoxic CD8 + T cells tended to decrease. In summary, it is apparent that chemotherapy exerts an influence on the tumor microenvironment of osteosarcoma, potentially by regulating the composition of immune cells. Notably, a marked decrease in the frequency of CD16 + NK cells and CD8 + T cytotoxic cells was noted, which may have contributed to an augmented antitumor response.

Research supports the pivotal role of osteoclast-mediated bone resorption in bone remodeling within primary osteosarcoma (Yin et al. 2016). Zhou's study also confirmed that osteoclasts are necessary and beneficial for the growth and metastasis of osteosarcoma (Zhou et al. 2020). These findings indicate the significant role of osteoclasts in promoting the malignant biological behaviors of osteosarcoma cells. Our results further suggest that chemotherapy may enhance the infiltration and maturation of osteoclasts, potentially affecting the tumor microenvironment of osteosarcoma through bone remodeling. Previous research has shown that chemotherapy can suppress ovarian function in breast cancer patients, leading to a rapid decrease in estrogen levels, disrupting the balance of bone resorption and formation, and resulting in osteoporosis (Vehmanen et al. 2001). However, chemotherapy-induced bone loss is not exclusively related to ovarian dysfunction in patients. Postmenopausal estrogen production occurs in peripheral fat, not in the ovaries, but chemotherapy can also lead to a decrease in bone density. Therefore, the enhanced activity of osteoclasts may result from multiple factors, including endocrine factors and chemotherapy.

Nonetheless, this study has limitations, particularly due to the rarity of osteosarcoma and the specificity of clinical treatments. First, due to the high costs associated with single-cell sequencing technology and the uncommon nature of osteosarcoma, the sample size analyzed in this study was limited, potentially introducing selection bias due to the small sample size. Future research in this area will require multicenter collaboration and data sharing to overcome these challenges. Second, the post-chemotherapy osteosarcoma samples included in this study were collected within one to two months after chemotherapy, reflecting only the short-term effects of chemotherapy on the tumor microenvironment, not its long-term impacts. This limitation is dictated by the clinical treatment protocol of surgical resection following neoadjuvant chemotherapy for osteosarcoma. Moreover, the unique characteristics of osteosarcoma samples make spatial transcriptomics sequencing exceedingly difficult, with no related data currently available. Single-cell transcriptomics lacks the spatial context of the cells, potentially leading to false-positive findings, particularly in the analysis of intercellular signaling networks. Subsequent research should investigate the potential changes in the spatial niche of OS caused by chemotherapy, with a focus on spatial aspects.

## Materials and methods

### Patients and sample collection

This study received approval from the Ethics Committee of Cancer Hospital, Chinese.

Academy of Medical Sciences, and Peking Union Medical College (NCC2021C-232).

Single-cell RNA sequencing data (GSE162454 (Liu et al. 2021) and GSE152048 (Zhou et al. 2020)) were obtained from GEO database (https://www.ncbi.nlm.nih.gov/geo/). Osteosarcoma tissue used for IHC (n = 10), and IF (n = 3) were obtained from National Cancer Center.

### Preprocessing of scRNA-seq data

ScRNA-seq data analyses were conducted using the Seurat package (Hao et al. 2021) (version 4.4.0) in R (version 4.1.2, The R Foundation). Quality controls were implemented by retaining non ribosomal genes detected in at least 0.1% of all cells, cells with a minimum of 200 features, and cells with mitochondrial fraction below 10%. Doublets were identified using the DoubletFinder (McGinnis et al. 2019) package (version 2.0.3) and subsequently removed. Normalization of raw unique molecular identifier (UMI) counts was carried out using the SCTransform function, with the number of cells set to 3,000. Harmony package (Korsunsky et al. 2019) (version 1.1.0) was employed to adjust for possible batch effects arising from patient-specific expression patterns. Dimension reduction was performed through principal-component analysis (PCA) using the RunPCA function, and the optimal number of principal components (PCs) was determined using the ElbowPlot function. The same PCs were applied in cell clustering with modularity optimization using the kNN graph algorithm as input. Visualization of cell clusters was achieved using the UMAP algorithm.

### Cell type annotation

Cell types were annotated by assessing the expression of known marker genes. Specifically, cells expressing marker genes from at least two types of major cell types were considered undefined cells and were consequently excluded from further analysis. Cell subtypes were annotated through unsupervised clustering and examination of marker gene expression levels, as depicted in corresponding figures. Differential expression genes (DEGs) within each cell subcluster were identified using the "FindAllMarker" function with default parameters provided by Seurat. These DEGs played a crucial role in cell type annotations, where cell subclusters exhibiting similar gene expression patterns were annotated as the same cell type.

### Trajectory analysis by Monocle 2 analysis

For trajectory analysis using Monocle 2 (Cao et al. 2019). First, the DEGs of different cell subclusters were identified and subjected to Monocle analysis. Then, dimensionality reduction and visualization were performed using “DDRTree” and “plot_cell_trajectory” functions in the Monocle 2 package. To visualize the expression of individual genes along pseudotime, a heatmap was generated using the "plot_pseudotime_heatmap" function.

### Pathway analysis

Differentially expressed genes (DEGs) meeting the criteria of |logFC|> 0.5 and an adjusted P value < 0.05 were utilized for Gene Ontology (GO) enrichment analysis. The "compareCluster" function from the clusterProfiler package (Wu et al. 2021) was applied to identify significantly enriched GO terms that exhibited differences between distinct fibroblast subclusters. To evaluate the differences in pathways across distinct subsets, Gene Set Variation Analysis (GSVA) and Gene Set Enrichment Analysis (GSEA) were performed. These analyses were conducted and computed using a linear model provided by the limma package.

### Single-cell regulatory network analysis

In accordance with a standardized analysis pipeline, we employed PySCENIC (Sande et al. 2020) (https://github.com/aertslab/SCENIC) to examine differentially expressed transcription factors (TFs) and delve into the single-cell gene regulatory network (GRN) within distinct cell subclusters. To outline the process, we generated gene expression matrices for the cell subclusters using GENIE3, establishing initial coexpression GRNs. Subsequently, the RcisTarget package was employed to identify TF motifs within the regulon data. The AUCell package was utilized to calculate the regulon activity score for each cell. Lastly, we filtered regulons with a correlation coefficient exceeding 0.3 with at least one other regulon, opting for those activated in a minimum of 30% of the cell subclusters for subsequent visualization.

### Differentiation of tumor cells and nonmalignant cells based on inferCNV

To distinguish tumor cells from nonmalignant cells, we employed the inferCNV R package (https://github.com/broadinstitute/inferCNV) to estimate the initial copy number variation (CNV) signal for each of the 60 regions using default parameters. The CNV signal was computed as the quadratic sum of the CNV region, and cells with a CNV signal surpassing 0.04 were designated as potential tumor cells in the context of OS (osteosarcoma).

### Gene set variation analysis

Pathway analyses were predominantly carried out on specific GO/Hallmark pathways obtained from the Molecular Signature Database (MSigDB version 6.2). To gauge pathway activity at the individual cell level, we utilized gene set variation analysis (GSVA) with default settings, implemented through the GSVA package (version 1.34.0).

### Gene set signature scoring

To quantitatively represent each cell subtype, we determined a subtype signature score, defined as the arithmetic mean of the expression levels of signature genes sourced from the specified data slot (e.g., SCT data slot). This methodology facilitates a standardized and comparative analysis of cell subtypes across various conditions and studies. Signature scores were computed using AUCell with default parameters (Aibar et al. 2017). The genes employed for gene set signature scoring are detailed in Supplementary Table 2.

### Western blot

All cell lines used for western blot were provided by the national cancer center. To perform a Western blot assay, total protein was extracted from cell lysate of cell lines, MCF7, MB-231, hFOB1.19, 143B, U2OS, HOS, MG63, and Saos2. All cell lines were authenticated by DNA fingerprinting analysis and tested free of mycoplasma infection. The proteinlevel was determined using a BCA kit. The proteins were then subjected to SDS-PAGE, transferred to a PVDF membrane, and probed with various antibodies from Abcam or for Proteintech specific proteins like OPN, Vinculin, SOX9, HIF1A, CD16, etc. After incubation with primary and secondary antibodies, the membrane was visualized using a chemiluminescent substrate in an imager.

### Immunofluorescence analysis

For immunohistochemistry and immunofluorescence, we collect tissue from areas of the tumor without obvious necrosis, approximately 1 × 1 × 1 cm in size. Tissue sections (n = 3) were deparaffinized, rehydrated, and subjected to antigen retrieval. After blocking non-specific binding with a suitable blocking agent, sections were incubated with primary antibodies overnight at 4 °C. Following washing, fluorescentlylabeled secondary antibodies were applied. Nuclei were stained with DAPI. Slides were mounted and images captured using a fluorescence microscope. Controls included sections incubated without primary antibodies to assess non-specific binding of secondary antibodies.

### Immunohistochemistry analysis

Tissue sections (n = 5 per group) were deparaffinized and rehydrated through graded alcohols to water. Antigen retrieval was performed using citrate buffer in a microwave. Endogenous peroxidase activity was quenched with hydrogen peroxide. After blocking with normal serum, sections were incubated with primary antibodies overnight at 4 °C. Biotinylated secondary antibodies were applied, followed by amplification with an avidinbiotin complex. Color development was achieved with DAB substrate. Sections were counterstained with hematoxylin, dehydrated, and mounted. Controls omitted the primary antibody. Wilcoxon test was utilized to compare the IF cores between pre- and post-chemotherapy samples.

### Statistical analysis

The in the study were conducted using R 4.3.0 and Python 3.9.0. Specific statistical details and methods are described in the figure legends, main text, or methods section. P-values were calculated using a two-sided, unpaired Wilcoxon rank-sum test. For error representation, either standard error of the mean (S.E.M.) or standard deviation (S.D.) was used, based on a minimum of three independent experiments.

## Conclusion

In summary, our study presents the comprehensive transcriptional landscape of tumor ecosystem remodeling induced by chemotherapy in osteosarcoma. We offer a detailed depiction of the shift from antitumor to protumor programs, characterized by an increase in cancer-associated fibroblasts (CAFs) and a reduction in immune cell populations, such as T/NK and myeloid cells. Within this altered landscape, we identified novel key players post-chemotherapy. Specifically, we observed that chemotherapy resulted in a subpopulation of osteosarcoma (OS) cells with upregulated expression of genes encoding secreted factors, such as SPP1 and LUM, as well as a distinct population of malignant OS cells. Furthermore, we demonstrated that chemotherapy diminishes antitumor immune responses, hindering the recruitment of CD16 + NK cells and cytotoxic CD8 + T cells. Moreover, chemotherapy enhances the infiltration of CAFs and their capacity to remodel the extracellular matrix (ECM), which may play a pivotal role in the therapeutic response. We acknowledge the need for studies with larger sample sizes to validate our findings and further explore cell subclusters associated with treatment responses. In conclusion, our work offers unprecedented insights into the remodeling of the tumor ecosystem induced by chemotherapy, which could inform and enhance treatment strategies for osteosarcoma.

### Supplementary Information

Below is the link to the electronic supplementary material.

Supplementary file1 (PDF 1400 KB)

Supplementary file2 (PDF 4245 KB)

Supplementary file3 (PDF 4356 KB)

Supplementary file4 (PDF 3920 KB)

Supplementary file5 (PDF 2549 KB)

Supplementary file6 (PDF 5427 KB)

Supplementary file7 (PDF 1145 KB)

Supplementary file8 (DOCX 18 KB)

Supplementary file9 (XLSX 10 KB)

## Data Availability

The datasets used are publicly available in GSE162454 and GSE15204855, all codes are available from the corresponding author on reasonable request.
