# Supplementary figures and images for: Single-cell transcriptomic insights into chemotherapy-induced remodeling of the osteosarcoma tumor microenvironment
Source: J Cancer Res Clin Oncol. 2024 Jul 20;150(7):356. doi: 10.1007/s00432-024-05787-2 (PMC11271355; doi:10.1007/s00432-024-05787-2)

A

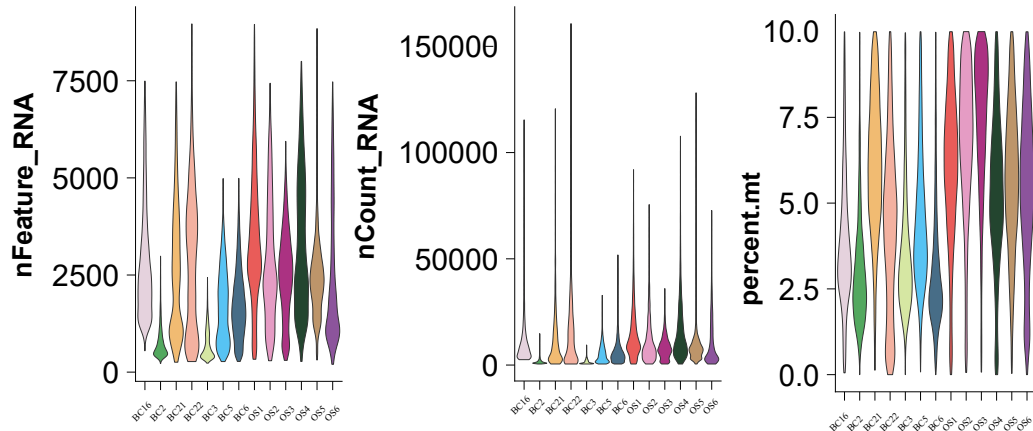

B

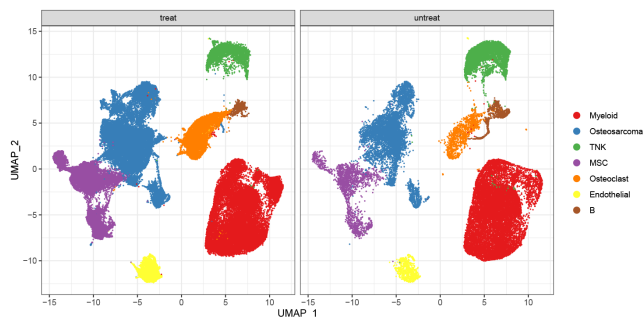

C

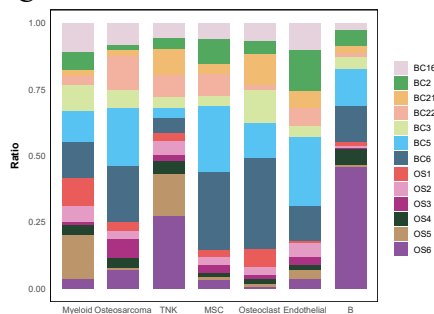

D

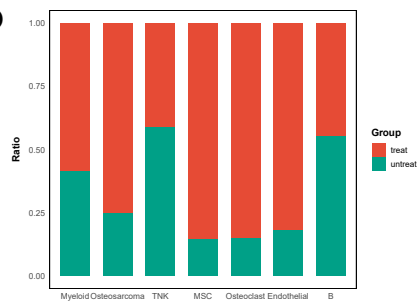

E

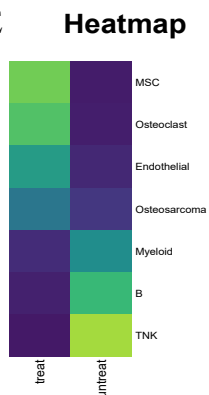

F

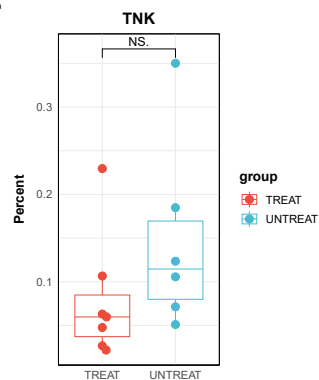

Supplement: Supplementary file 1 — Supplementary file1 (PDF 1400 KB) [file 432_2024_5787_MOESM1_ESM.pdf]

A

Total = 24890

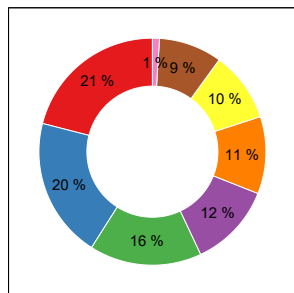

Celltype

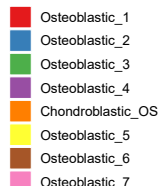

B

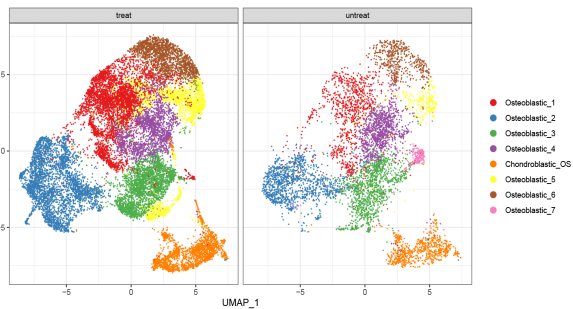

C

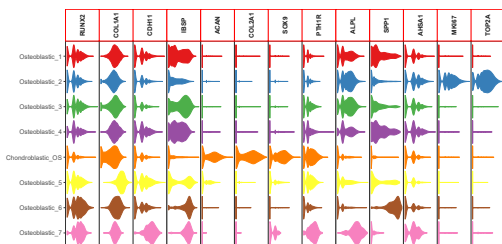

D

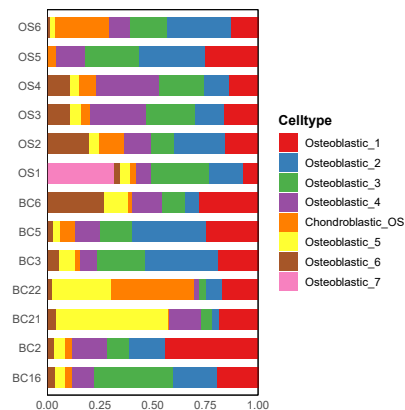

E

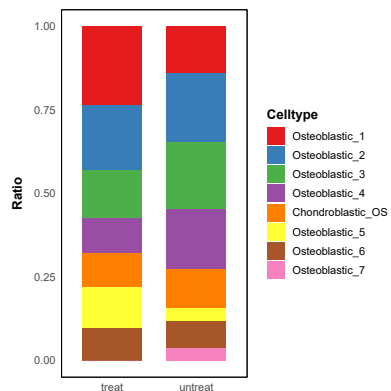

H

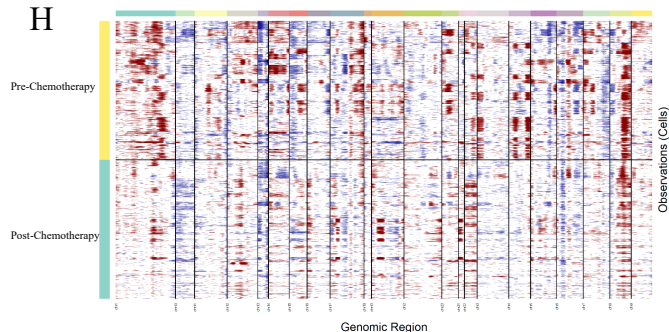

F

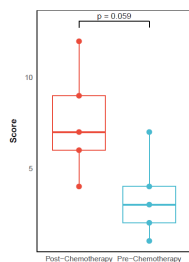

G

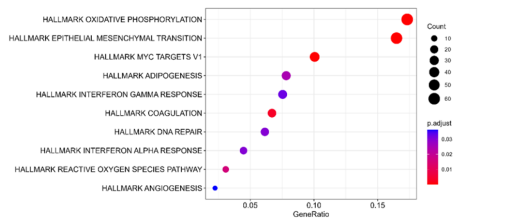

Supplement: Supplementary file 2 — Supplementary file2 (PDF 4245 KB) [file 432_2024_5787_MOESM2_ESM.pdf]

A

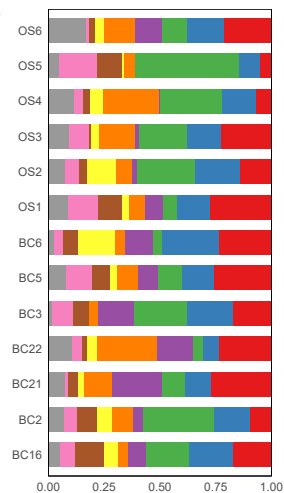

B

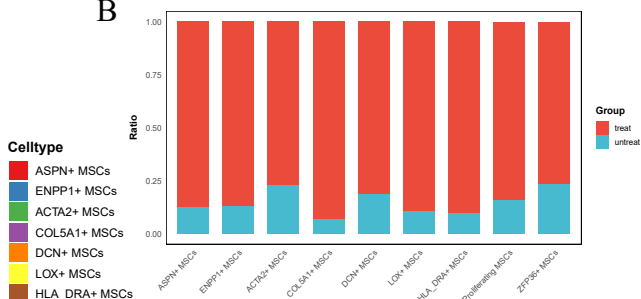

C

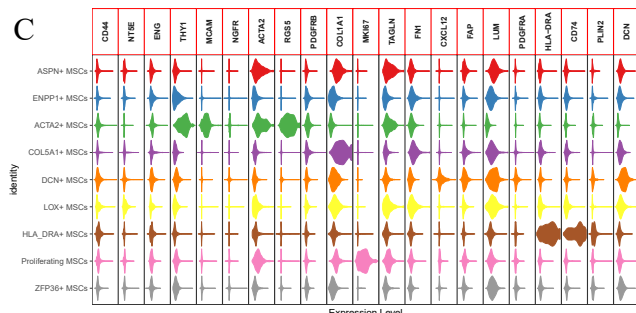

D

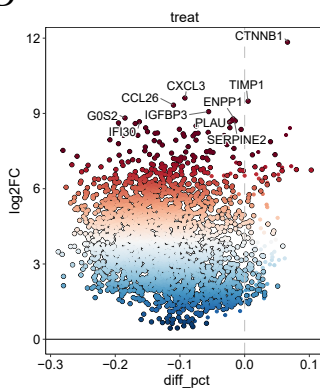

E

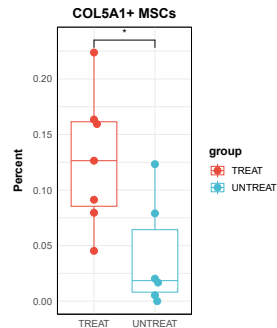

F

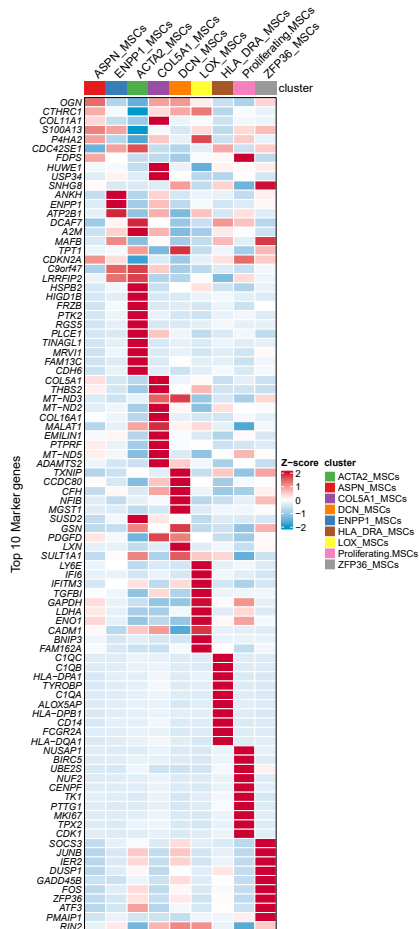

Supplement: Supplementary file 3 — Supplementary file3 (PDF 4356 KB) [file 432_2024_5787_MOESM3_ESM.pdf]

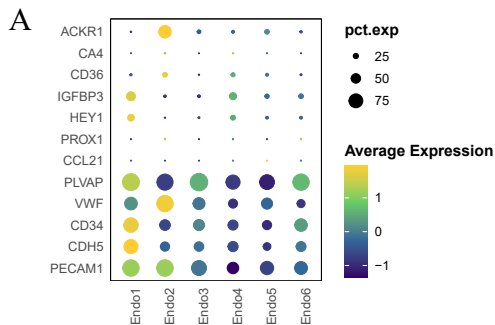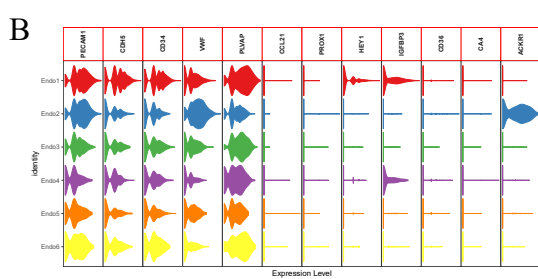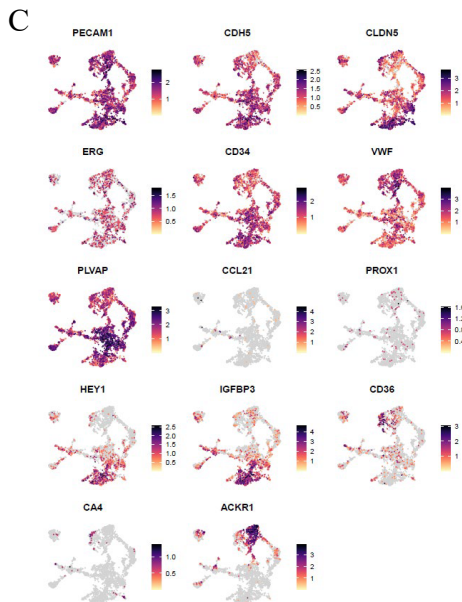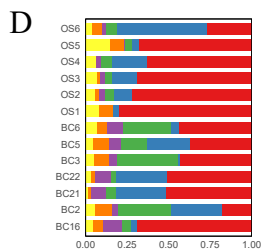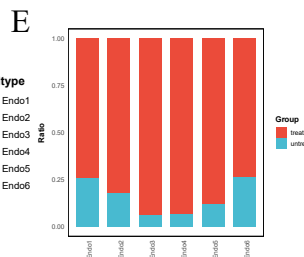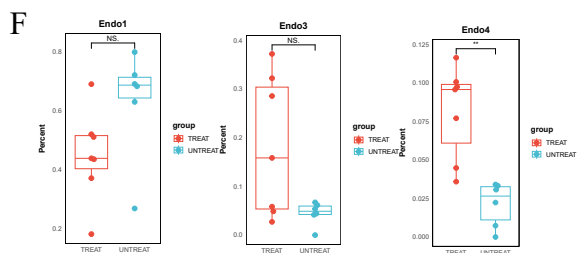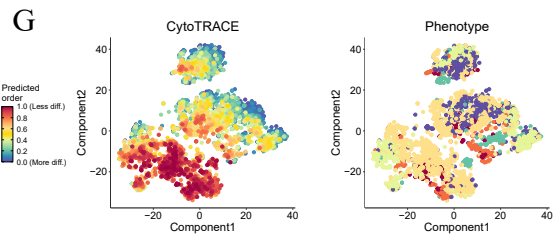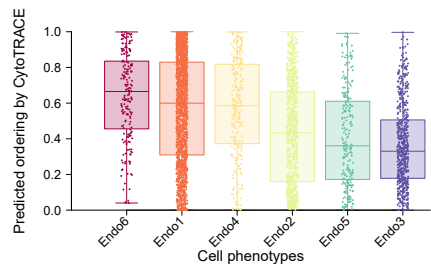

Supplement: Supplementary file 4 — Supplementary file4 (PDF 3920 KB) [file 432_2024_5787_MOESM4_ESM.pdf]

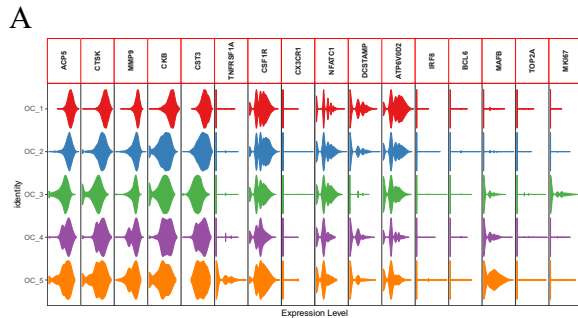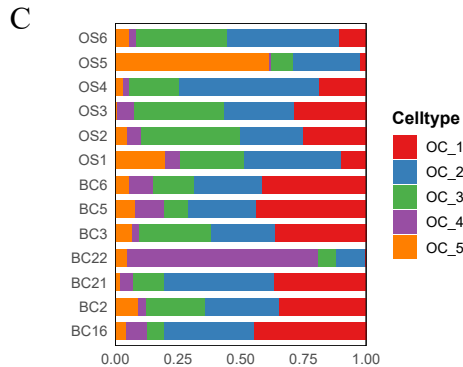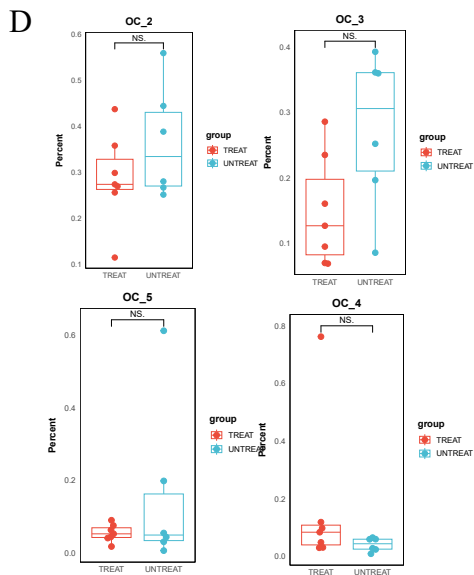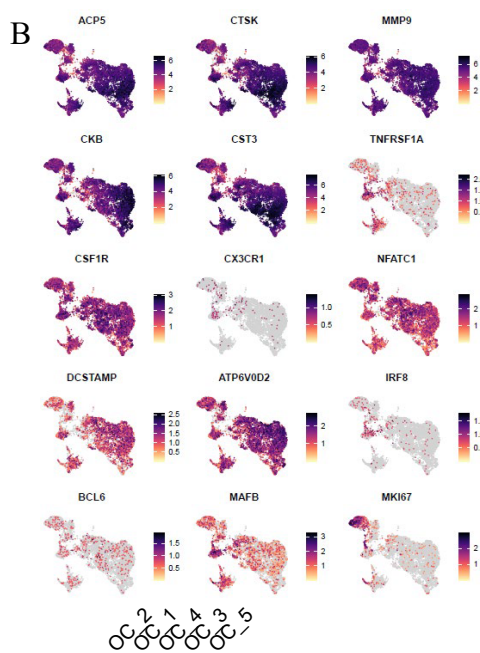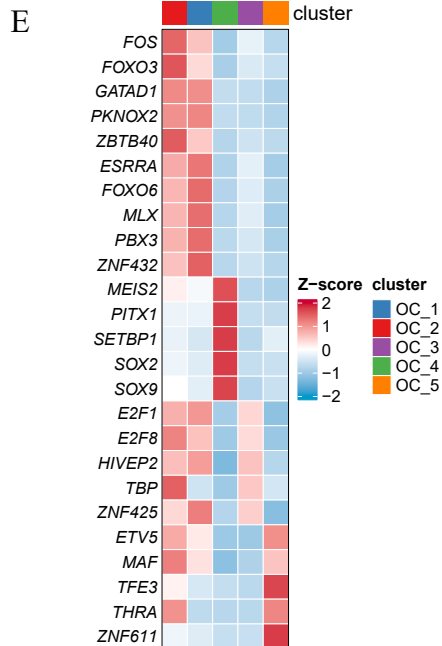

Supplement: Supplementary file 5 — Supplementary file5 (PDF 2549 KB) [file 432_2024_5787_MOESM5_ESM.pdf]

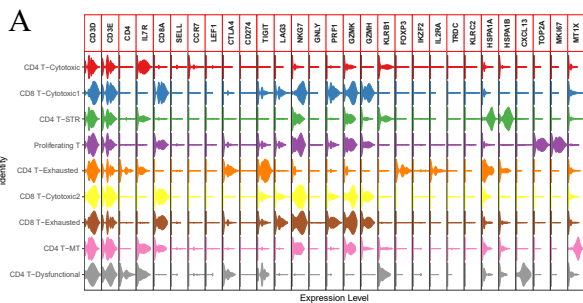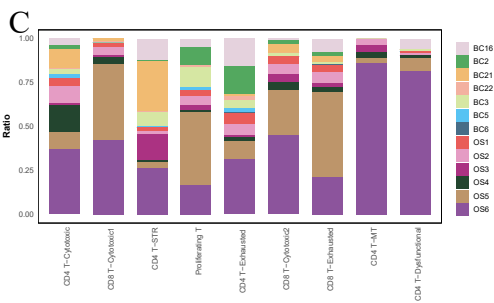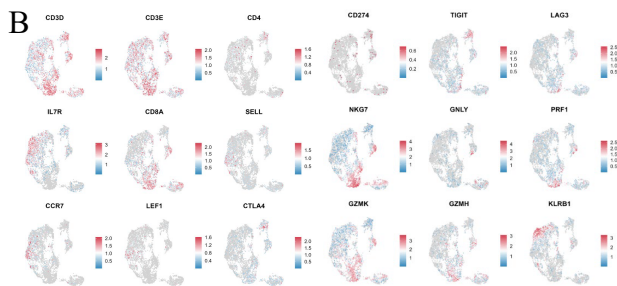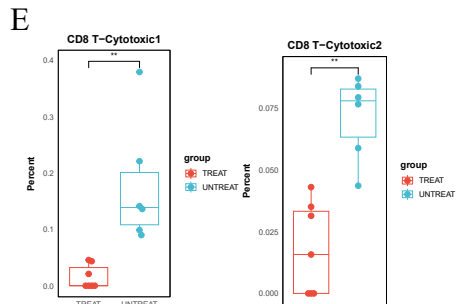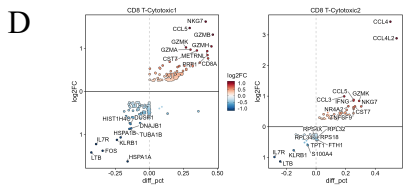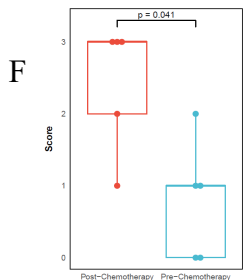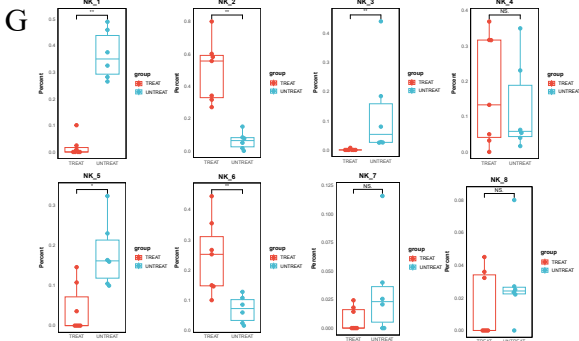

Supplement: Supplementary file 6 — Supplementary file6 (PDF 5427 KB) [file 432_2024_5787_MOESM6_ESM.pdf]

A

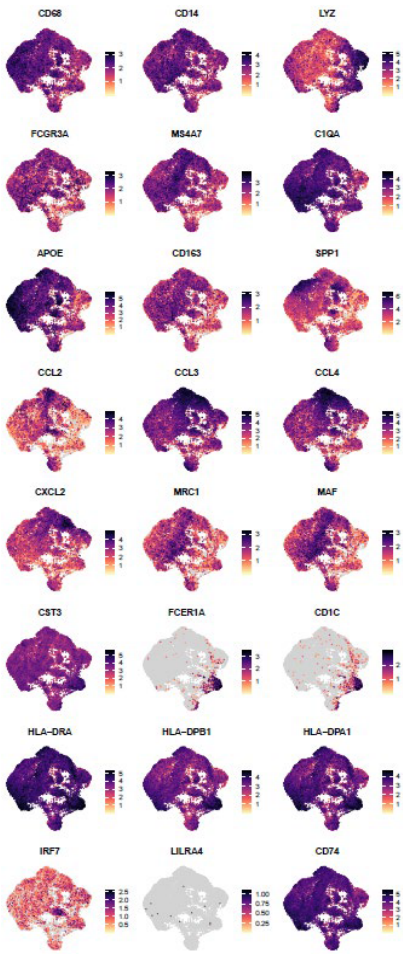

B

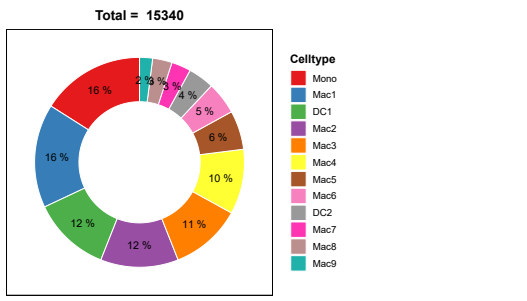

C

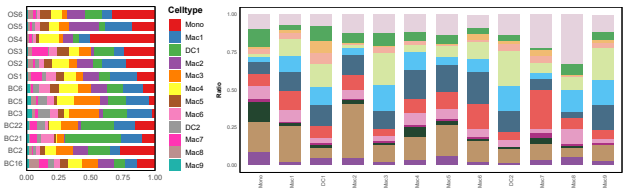

D

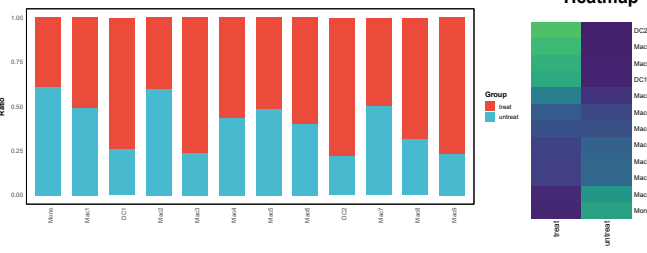

Supplement: Supplementary file 7 — Supplementary file7 (PDF 1145 KB) [file 432_2024_5787_MOESM7_ESM.pdf]
