# Supplementary material for: Single-cell transcriptomic insights into chemotherapy-induced remodeling of the osteosarcoma tumor microenvironment
Source: J Cancer Res Clin Oncol. 2024 Jul 20;150(7):356. doi: 10.1007/s00432-024-05787-2 (PMC11271355; doi:10.1007/s00432-024-05787-2)
Supplement: Supplementary file 8 — Supplementary file8 (DOCX 18 KB) [file 432_2024_5787_MOESM8_ESM.docx]

Table1 Clinical characteristics of the 23 patients with OS included in this study

| Sample | Pathological type | Type | Location | Pre- or Post-Chemotherapy | Data Source |
| --- | --- | --- | --- | --- | --- |
| BC2 | Conventional | Primary | Femur | Post-Chemotherapy | GSE152048 |
| BC3 | Conventional | Primary | Tibia | Post-Chemotherapy | GSE152048 |
| BC5 | Conventional | Primary | Femur | Post-Chemotherapy | GSE152048 |
| BC6 | Conventional | Primary | Ulna | Post-Chemotherapy | GSE152048 |
| BC16 | Conventional | Primary | Tibia | Post-Chemotherapy | GSE152048 |
| BC21 | Intraosseous  osteosarcoma | Primary | Femur | Post-Chemotherapy | GSE152048 |
| BC22 | Chondroblastic | Primary | Femur | Post-Chemotherapy | GSE152048 |
| OS1 | Classical osteosarcoma | Primary | NA | Pre-Chemotherapy | GSE162454 |
| OS2 | Classical osteosarcoma | Primary | NA | Pre-Chemotherapy | GSE162454 |
| OS3 | Classical osteosarcoma | Primary | NA | Pre-Chemotherapy | GSE162454 |
| OS4 | Classical osteosarcoma | Primary | NA | Pre-Chemotherapy | GSE162454 |
| OS5 | Classical osteosarcoma | Primary | NA | Pre-Chemotherapy | GSE162454 |
| OS6 | Classical osteosarcoma | Primary | NA | Pre-Chemotherapy | GSE162454 |
| BS1 | Classical osteosarcoma | Primary | Tabia | Pre-Chemotherapy | National Cancer Center |
| BS2 | Classical osteosarcoma | Primary | Ilium | Pre-Chemotherapy | National Cancer Center |
| BS4 | Classical osteosarcoma | Primary | Femur | Pre-Chemotherapy | National Cancer Center |
| BS6 | Classical osteosarcoma | Primary | Femur | Pre-Chemotherapy | National Cancer Center |
| BS8 | Classical osteosarcoma | Primary |  | Pre-Chemotherapy | National Cancer Center |
| BS3 | Classical osteosarcoma | Primary | Humerus | Post-Chemotherapy | National Cancer Center |
| BS5 | Classical osteosarcoma | Primary | Femur | Post-Chemotherapy | National Cancer Center |
| BS7 | Classical osteosarcoma | Primary | Tabia | Post-Chemotherapy | National Cancer Center |
| BS9 | Classical osteosarcoma | Primary | Femur | Post-Chemotherapy | National Cancer Center |
| BS15 | Classical osteosarcoma | Primary | Tabia | Post-Chemotherapy | National Cancer Center |

GSE162454 and GSE152048 were downloaded from GEO database, and others including BS1/ BS2/ BS4/ BS6/ BS8/ BS3/ BS5/ BS7/ BS9/ BS15 were collected for National Cancer Center.
